# The impact of AI precision feedback on college students’ thinking shaping ability: mediating effect of intrinsic value identification and moderating role of critical consciousness transformation

**DOI:** 10.3389/fpsyg.2026.1798182

**Published:** 2026-04-15

**Authors:** Wenfei Sun, Qingqing Yuan, Qiancheng Zhou

**Affiliations:** 1 School of Marxism, Sichuan University, Chengdu, China; 2 College of Education, Zhejiang University, Hangzhou, China; 3 School of Public Administration, Jilin University, Changchun, China

**Keywords:** AI precision feedback, college students, critical consciousness transformation, intrinsic value identification, moderated mediation model, thinking shaping ability

## Abstract

**Background:**

In the context of educational digital transformation, AI precision feedback has emerged as a critical technological support for optimizing college students’ cognitive development. As a key dimension of core competencies, thinking shaping ability directly impacts academic progress and lifelong learning capabilities.

**Methods:**

To elucidate the mechanism of AI precision feedback in shaping students’ thinking shaping ability, this study conducted a mediation analysis with 1,079 college students using path analysis, Bootstrap sampling, and moderation testing. The research constructed a mediation model: “AI precision feedback → intrinsic value identification → thinking shaping ability,” incorporating critical consciousness transformation as a moderating variable.

**Results:**

Results revealed: (1) AI precision feedback significantly enhances thinking shaping ability (standardized coefficient = 0.561, *p* < 0.001); (2) Intrinsic value identification partially mediates this relationship [mediation effect = 0.153, accounting for 32.01% of total effect; 95% Bootstrap CI = (0.061, 0.284)]; (3) Critical consciousness transformation significantly moderates the relationship between intrinsic value identification and college students’ thinking shaping ability (interaction ^**^p = 0.040), and the transformation of high critical consciousness can strengthen the promoting effect of intrinsic value identification on thinking shaping ability.

**Conclusion:**

This study enriches the theoretical framework for AI education and cognitive cultivation, providing empirical evidence and practical guidance for universities to optimize AI technology applications and precisely enhance students’ cognitive literacy.

## 1 Introduction

Amid the digital transformation wave driven by AI technology’s deep integration into education, teaching models are rapidly evolving from traditional “knowledge cramming” to “precision empowerment” (Doyle et al., 2025). AI precision feedback, with its data-driven personalization, real-time interaction, and targeted improvement suggestions, overcomes the limitations of traditional education feedback such as delays and generalization (Guo et al., 2024). It provides customized guidance for college students’ learning processes, becoming a key driver for enhancing higher education quality (Liu and Deris, 2025). College students are at a critical stage of thinking shaping ability (Huang et al., 2026). The ability to shape thinking, as a core indicator of thinking shaping ability, encompasses multiple dimensions including logical reasoning, innovative thinking, and problem-solving. This ability directly impacts academic research outcomes, career potential, and lifelong learning capabilities, making it one of the core objectives in higher education talent cultivation (Lu, 2025). Currently, global higher education is undergoing profound digital transformation. According to the “2023 Horizon Report: Education and Learning Edition,” artificial intelligence has become the primary technological trend influencing the future development of higher education. In China, the Ministry of Education’s “Education Informatization 2.0 Action Plan” explicitly proposes to promote the deep integration of artificial intelligence with teaching and learning. By 2024, over 80% of universities nationwide had introduced intelligent teaching platforms or AI-assisted learning systems. However, a significant gap remains between technological application and educational effectiveness: on one hand, intelligent feedback systems are widely deployed; on the other hand, their actual impact on students’ higher-order thinking skills (such as critical thinking and innovative thinking) lacks systematic validation. This misalignment between “technological investment” and “capacity output” constitutes the core realistic context of this study. In this context, exploring the intrinsic connection between AI precision feedback and college students’ thinking shaping ability, analyzing its operational mechanisms and boundary conditions, holds significant theoretical and practical value for optimizing AI education applications and enhancing students’ core competencies.

From a practical development perspective, universities currently face a dual dilemma. First, large-scale enrollment expansion has led to a continuous decline in the student-to-faculty ratio, making it difficult for teachers to provide timely and personalized feedback on cognitive training for each student. Statistics from the Ministry of Education show that the student-to-faculty ratio in regular higher education institutions nationwide reached 17.5:1 in 2023, with some popular majors even exceeding 20:1. The traditional manual feedback model has become unsustainable in terms of coverage and timeliness. Second, the widespread adoption of generative artificial intelligence (such as ChatGPT and DeepSeek) is reshaping knowledge acquisition methods, posing real challenges for college students regarding “declining cognitive abilities” and “AI dependency risks.” The “2024 China College Students’ Generative AI Usage Survey Report” reveals that 67.4% of students use generative AI tools more than three times per week, with 23.8% admitting to “directly copying AI answers without critical thinking.” Universities urgently need to leverage AI technology to achieve “precision empowerment,” but there is a prevalent tendency in practice to “prioritize technology over education”—existing intelligent systems often focus on correct/incorrect knowledge point judgments, lacking in-depth feedback on cognitive processes (such as logical flaw analysis and innovative path guidance). This inherent contradiction constitutes the direct source of issues in this study.

As artificial intelligence (AI) finds increasingly widespread application in educational settings, research has demonstrated that intelligent feedback technology can effectively enhance students’ learning efficiency and knowledge mastery (Naseer and Khawaja, 2025; Nazaretsky et al., 2024). For instance, smart tutoring systems can pinpoint knowledge gaps through precise analysis of students’ learning behavior data, providing personalized error correction suggestions to help students optimize their learning strategies (Milička et al., 2025). However, existing studies predominantly focus on the impact of AI feedback on explicit outcomes such as academic performance and knowledge acquisition, while insufficient exploration exists regarding its underlying mechanisms in shaping implicit cognitive abilities. The development of thinking shaping ability constitutes a complex internal psychological process, influenced not only by direct external educational interventions but also indirectly through individual psychological and cognitive mediators (Xu et al., 2023). Internal value identification, representing subjective acceptance and meaning attribution of external interventions, reflects college students’ recognition of AI’s precision feedback and willingness to actively utilize it (Yu et al., 2025). Whether this internal value identification can serve as a bridge between intelligent feedback and thinking shaping ability remains a critical question worthy of in-depth investigation.

It is noteworthy that the current evaluation system for AI-powered education applications remains underdeveloped. Existing assessments predominantly focus on explicit indicators such as academic performance and homework completion rates, while significantly lacking measurement and tracking of implicit competencies like critical thinking development. Although the OECD’s PISA 2022 Creative Thinking Assessment Report has incorporated creative thinking into international evaluation frameworks, domestic universities still lack systematic mechanisms and validation of effectiveness in utilizing AI technology to cultivate higher-order thinking skills. This evaluation gap makes it challenging to accurately measure the true impact of AI education applications, thereby limiting the precision of technological optimization and pedagogical improvement efforts. Meanwhile, college students are not passive recipients of feedback generated by artificial intelligence. Critical consciousness transformation, as an individual’s ability to filter, question, integrate, and apply external information, helps them rationally evaluate the advantages and limitations of AI feedback, avoiding blind reliance or excessive rejection. In today’s era of information explosion and rapid technological iteration, there are significant individual differences in students’ critical consciousness transformation levels (Alwafi, 2025; Chen et al., 2025). These differences may affect their implementation of intrinsic value identification, thereby regulating the relationship between intrinsic value identification and thinking shaping ability (Furey et al., 2025). Currently, research on the moderating role of critical consciousness transformation in AI education applications remains scarce, lacking systematic theoretical understanding and empirical support.

A comprehensive analysis of the policy context, current application status, practical challenges, and evaluation gaps reveals that current research still lacks comprehensive exploration of the impact mechanisms, mediating pathways, and moderating factors of AI precision feedback on college students’ thinking shaping ability. To address these gaps, this study constructs a mediation model examining the relationship between AI precision feedback, intrinsic value identification, and thinking shaping ability, with critical consciousness transformation serving as a moderating variable. The research focuses on three key questions: (1) Whether AI precision feedback significantly positively correlates with college students’ thinking shaping ability; (2) Whether intrinsic value identification mediates the relationship between AI precision feedback and college students’ thinking shaping ability; (3) Whether critical consciousness transformation moderates the effect of intrinsic value identification on college students’ thinking shaping ability. Through empirical analysis, this study aims to reveal complex interrelationships among variables, providing scientific evidence and practical guidance for refining AI education frameworks and optimizing cognitive development pathways for college students.

## 2 Theoretical review and literature summary

### 2.1 AI precision feedback

With the rapid advancement of artificial intelligence technology, its applications in education have become increasingly widespread and profound, with AI precision feedback emerging as a key research focus (Li et al., 2025). Numerous studies demonstrate that AI precision feedback systems offer significant advantages over traditional methods, effectively overcoming limitations in conventional feedback approaches (Navio-Ingles et al., 2025). Traditional feedback mechanisms are often constrained by teachers’ time and energy, making it challenging to provide comprehensive, detailed, and timely feedback for every student (Pears et al., 2025). In contrast, AI leverages its robust data processing capabilities to collect and analyze students’ learning data in real-time, covering multiple aspects such as learning behaviors, test-taking performance, and knowledge mastery levels. This enables the delivery of precise, personalized feedback tailored to individual needs (Preda-ulita, 2025). For instance, some intelligent learning systems can accurately identify students’ knowledge gaps based on error patterns in their test answers, then recommend targeted learning materials and practice questions to help students effectively address knowledge deficiencies and enhance learning outcomes. Such precision feedback not only helps students promptly understand their academic progress but also motivates them to engage more actively in their learning process (Steiss et al., 2024; Yan, 2025).

From the theoretical evolution of feedback mechanisms, AI-powered precision feedback represents a paradigm shift in educational feedback from “delayed-unified” to “real-time-personalized” approaches. The feedback model proposed by Hattie and Timperley emphasizes that “feedback answers three fundamental questions: Where am I headed? How do I get there? What’s next?” AI precision feedback extends this theory through data mining technologies. Specifically, its accuracy manifests in three dimensions: content precision (identifying specific cognitive gaps using knowledge graphs), timing precision (predicting optimal intervention points based on learning curves), and method precision (personalized presentation formats tailored to learner profiles). Recent research has further categorized AI feedback into three tiers: first-level outcome feedback (correct/incorrect judgments), second-level process feedback (error type diagnosis), and third-level metacognitive feedback (strategic recommendations and cognitive monitoring). This study focuses on the profound impact of third-level feedback on cognitive shaping capabilities, a feedback mechanism that remains inadequately explored in existing literature.

### 2.2 College students’ thinking shaping ability

The ability of college students’ thinking shaping is the key component of their comprehensive quality, which plays a vital role in their future development (Daner et al., 2024). The ability of shaping thinking includes many kinds of thinking quality, such as critical thinking, creative thinking, logical thinking, etc. In the knowledge economy era, society demands higher cognitive abilities from university students, requiring not only solid academic foundations but also the capacity for independent thinking and innovative problem-solving (Sohail et al., 2024; Xavier et al., 2025). Research indicates that the university years constitute a critical phase for thinking shaping ability (Rad et al., 2024). Through scientific educational approaches and guidance, students’ thinking skills can be effectively enhanced. For instance, implementing inquiry-based and project-based learning models encourages active participation in discussions, practical activities, and explorations, thereby fostering critical and creative thinking (Guo et al., 2025; Kanel et al., 2019; Luna et al., 2021). Moreover, a supportive learning environment and diverse academic activities provide ideal conditions for cognitive growth, stimulating intellectual vitality and broadening perspectives (Lim et al., 2026).

From a conceptual perspective, cognitive shaping ability differs from conventional thinking ability by emphasizing plasticity and developmental characteristics. This study employs Facione’s critical thinking cognitive skills framework and Guilford’s divergent thinking theory as theoretical foundations, operationalizing college students ‘cognitive shaping ability into four measurable dimensions: logical analysis skills (deductive and inductive reasoning), innovative generation capacity (conceptual fluency and adaptability), problem reconstruction capability (problem identification and strategy selection), and metacognitive monitoring skills (reflection and regulation of cognitive processes). This definition transcends previous research limitations that viewed thinking ability as static traits, highlighting the dynamic developmental process under educational interventions. Notably, cognitive ability development exhibits situational embeddedness—AI feedback environments, as novel learning contexts, may reshape traditional thinking training’s spatiotemporal structures through their real-time interaction features. The unique mechanism by which this “technological context” influences cognitive shaping constitutes the core issue this study aims to elucidate.

### 2.3 Intrinsic value identification

Intrinsic value identification plays a pivotal role in education and learning. It refers to an individual’s profound understanding and positive experience of learning activities and their significance, serving as the internal driving force that motivates students to engage in active learning (Guo et al., 2025; Aaron et al., 2025). When students exhibit high intrinsic value identification, they perceive learning as a process of self-improvement and self-actualization, leading them to participate more proactively and persist through challenges. Research indicates that intrinsic value identification is closely linked to students’ learning motivation, engagement, and outcomes (Choi, 2025). For instance, students with strong intrinsic value identification toward their major are more willing to invest time and effort in mastering professional knowledge, actively participate in practical activities, and achieve better academic performance (Bernik et al., 2025, Yin et al., 2025). In educational practice, teachers can enhance students’ intrinsic value identification by guiding them to recognize the value of learning and stimulating their interest, thereby promoting their academic growth and development (Hwang et al., 2023).

From the perspective of social cognitive theory, the formation of intrinsic value identification follows a progressive process of “information exposure → meaning construction → value internalization.” In AI education contexts, this process exhibits unique characteristics: First, the impersonal nature of AI feedback may weaken emotional connections in traditional teacher-student interactions while potentially reducing students ‘psychological defenses due to its de-evaluation anxiety effect. Second, the high-frequency iterative nature of AI feedback (instant error correction-immediate improvement) creates frequent opportunities for “success experiences,” which may accelerate the formation cycle of value identification. Third, the visual presentation of AI feedback (learning trajectory maps, competency radar charts) provides concrete representations for abstract learning values. Existing research has insufficient attention to value identification mechanisms in technology-mediated contexts, particularly lacking exploration of the relationship between “technology trust” and “value identification” —does students’ trust in the accuracy of AI feedback constitute a prerequisite for value identification? This study operationalizes intrinsic value identification into three components: utility identification (belief that AI feedback enhances learning outcomes), process identification (enjoyment of learning interactions with AI), and self-referential identification (integration of AI feedback into personal learning systems). This multidimensional framework better captures the complexity of value identification in AI environments.

### 2.4 Transformation of critical consciousness

The transformation of critical consciousness is an important link in the development of thinking, which reflects the ability of the individual to analyze, evaluate and judge the information by using critical thinking, and to transform this thinking into practical actions (Jin et al., 2025; Martín Lucas et al., 2025; Mariano et al., 2025). In this age of information explosion, students are constantly exposed to vast amounts of data (Dinh, 2025; Gendia, 2022). The ability to filter valuable information, engage in critical thinking, and apply it effectively is a hallmark of critical consciousness transformation (Brügge et al., 2024). Research shows that students with strong critical consciousness transformation skills excel at tackling complex learning tasks and real-world challenge. Rather than blindly accepting others’ opinions, they develop independent perspectives through critical analysis and reasoning (Borup et al., 2025; Darvishi et al., 2024). Cultivating this ability is essential in education. Teachers can stimulate critical thinking by guiding students to ask questions, participate in discussions, and analyze case studies, helping them view problems from multiple angles and enhance their problem-solving skills (Leong and Sung, 2025; Tan and Biswas, 2006).

The concept of Critical Consciousness Transformation originates from Freire’s liberative education theory, emphasizing a complete continuum from “consciousness awakening” to “action transformation.” In AI education contexts, this framework carries unique contemporary significance: students must develop algorithmic literacy to interpret algorithm-generated feedback, including understanding system logic, identifying potential biases, and evaluating the applicability of recommendations. Halpern’s critical thinking theory distinguishes between “skills” and “tendencies.” This study operationalizes critical consciousness transformation as an integration of cognitive skills (analysis, evaluation, and inference) and metacognitive tendencies (self-regulation of thought processes), with particular emphasis on transferability in technological environments. The core question is whether students can apply critical thinking skills to AI interactions—effectively selectively processing feedback and creatively transforming it. The theoretical rationale behind this regulatory mechanism lies in the following: Students with heightened critical awareness transform AI feedback from passive acceptance of “standard answers” into critical cognitive resources. They critically process such feedback by questioning its validity, integrating it with self-assessment, and generating original solutions that transcend conventional approaches. This process enhances the conversion of intrinsic value recognition into cognitive development capabilities. Conversely, students with low critical awareness may fall into extreme technological dependence or rejection, thereby diminishing the practical efficacy of value identification.

### 2.5 Summary of literature review

Current research has begun to explore the relationship between AI precision feedback, intrinsic value identification, critical consciousness transformation, and college students’ thinking shaping ability (Chen and Chen, 2026; Dann et al., 2024). Studies indicate that AI precision feedback enhances students’ thinking shaping ability by reinforcing their intrinsic value identification (Capecchi et al., 2025; Kamruzzaman et al., 2023). This targeted feedback allows learners to perceive tangible academic progress, strengthening their intrinsic motivation to engage more actively in learning activities and promote cognitive growth. However, research examining the mediating role of critical consciousness transformation between intrinsic value identification and thinking shaping ability remains limited (Rakhmetov et al., 2025; Suffian et al., 2022). While some studies acknowledge the importance of critical thinking in cognitive development, few delve into its specific regulatory mechanisms within variable relationships (Mi et al., 2025; Seneviratne et al., 2025). This study focuses on this gap by investigating how AI-based feedback influences thinking shaping ability, the mediating effect of intrinsic value identification, and the moderating role of critical consciousness transformation. The findings aim to provide targeted theoretical support and practical guidance for educational practices.

## 3 Research hypotheses and data sources

### 3.1 Research hypotheses

This study proposes the following hypotheses:

*Hypothesis 1*: AI precision feedback demonstrates a statistically significant positive correlation with college students’ college students’ thinking shaping ability.

*Hypothesis 2*: Intrinsic value identification plays a significant mediating role between AI precision feedback and college students’ thinking shaping ability.

*Hypothesis 3*: Critical consciousness transformation plays a moderating role between intrinsic value identification and college students’ thinking shaping ability.

**Figure 1:**
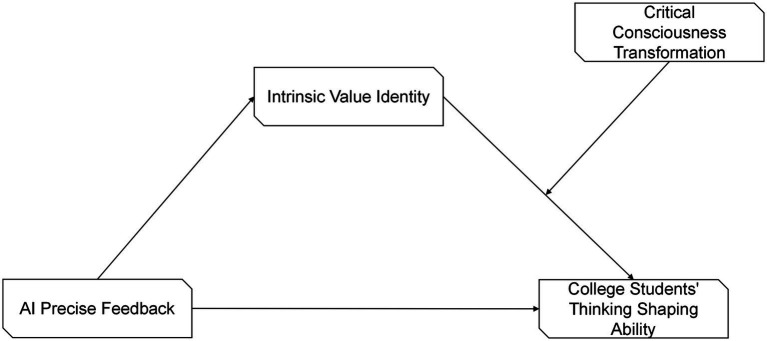
Research framework diagram.

The research framework of this paper is shown in Figure 1.

### 3.2 Data collection

To comprehensively and accurately investigate the impact of AI-powered feedback on college students’ thinking shaping ability, as well as the mediating role of intrinsic value identification and the moderating effect of critical consciousness transformation, this study employed a diversified data collection approach to ensure the richness, reliability, and validity of the data.

Questionnaire surveys serve as a cornerstone of data collection in this study. To ensure the quality and validity of the questionnaire, we conducted a pilot survey before formal distribution. Selected college students were invited to complete the questionnaire, and based on their feedback, we refined the questions by removing ambiguous or misleading items, optimizing phrasing and sequencing to enhance readability and ease of response. During the formal survey, we adopted a hybrid approach combining online and offline distribution. Online distribution utilized professional platforms like Wenjuanxing (a Chinese survey platform) and channels such as WeChat and QQ to expand reach. Offline distribution involved random paper questionnaire distribution in campus locations including teaching buildings, libraries, and cafeterias, ensuring data collection from students across different majors and academic years. This study collected 1,092 questionnaires, with 1,079 valid responses retained after removing invalid entries, ensuring sample representativeness and data sufficiency.

## 4 Research method

Path analysis, a multivariate statistical technique based on regression principles, investigates direct causal relationships among multiple variables. In this method, variable relationships are represented through path diagrams where arrows indicate causal directions and path coefficients reflect relationship strength and direction. This approach simultaneously considers the effects of multiple independent variables on dependent variables and their interrelationships, thereby comprehensively revealing complex variable interactions. In this study, path analysis primarily examines direct effect hypotheses to verify whether AI-precise feedback significantly positively correlates with college students’ thinking shaping ability. By constructing a path model with AI-precise feedback as the independent variable and thinking shaping ability as the dependent variable, we directly analyze their path coefficients. A significant positive path coefficient confirms direct positive influence of AI precise feedback on thinking shaping ability, validating *Hypothesis 1*. Path analysis clearly demonstrates direct causal pathways, providing robust statistical support for understanding the relationship between AI precise feedback and thinking shaping ability in college students.

Bootstrap sampling is a statistical inference method based on repeated sampling, which generates a large number of bootstrap samples by performing repeated sampling with replacement from the original sample. These bootstrap samples are then used to estimate statistics and conduct hypothesis testing. Compared to traditional parameter estimation methods, Bootstrap sampling does not rely on assumptions about the population distribution, enabling more accurate estimation of the sampling distribution of statistics. This provides unique advantages, especially when dealing with small sample sizes or unknown population distributions. In this study, Bootstrap sampling was primarily used to analyze mediating effects, specifically to verify whether intrinsic value identification plays a significant mediating role between AI precision feedback and college students’ thinking shaping ability. Testing mediating effects typically requires estimating the magnitude and significance of indirect effects. Bootstrap sampling can generate numerous indirect effect estimates through repeated sampling, thereby constructing confidence intervals for indirect effects. If the confidence interval does not contain 0, it indicates a significant indirect effect, meaning intrinsic value identification mediates the relationship between AI precision feedback and college students’ thinking shaping ability, thus validating *Hypothesis 2*. Bootstrap sampling provides more accurate estimates and test results for mediating effects, avoiding potential biases in traditional methods and enhancing the reliability of research conclusions.

Moderation effect refers to the ability of a variable (modulating variable) to influence the direction or strength of the relationship between an independent variable and a dependent variable. The modulation effect test aims to determine the role of the modulating variable in the relationship between the independent and dependent variables, typically achieved by introducing interaction terms between the independent variable and the modulating variable in the regression model. If the coefficient of the interaction term is significant, it indicates that the modulating variable moderates the relationship between the independent and dependent variables. In this study, the modulation effect test was primarily used to analyze the moderating effect, specifically to verify whether critical consciousness transformation moderates the relationship between intrinsic value identification and college students’ thinking shaping ability. By constructing a regression model that includes intrinsic value identification, critical consciousness transformation, and their interaction terms, we tested whether the interaction term coefficients were significant. If the interaction term coefficients were significant, it would suggest that critical consciousness transformation moderates the relationship between intrinsic value identification and college students’ thinking shaping ability. This implies that the impact of intrinsic value identification on thinking shaping ability varies across different levels of critical consciousness transformation, thereby validating *Hypothesis 3*. The modulation effect test enables in-depth exploration of complex variable relationships, reveals the mechanisms of modulating variables, and provides richer insights for comprehensively understanding the impact of AI precision feedback on college students’ thinking shaping ability.

## 5 Results

### 5.1 Sample size analysis

A sample size analysis was conducted on the 1,079 valid questionnaires collected for this study, with results presented in Table 1. The table displays basic demographic characteristics of the control variables, including gender, age, educational stage, and political affiliation.

**Table 1:** Descriptive statistics of sample size.

Category	Classification	Number of people	Percentage
Sex	Male	572	53.01%
	Female	507	46.99%
Age	18–22	468	43.37%
	23–26	351	32.53%
	27–30	182	16.87%
	31 and more	78	7.23%
Learning phase	Undergraduate	507	46.99%
	Master’s student	364	33.73%
	Doctoral student	208	19.28%
Political status	General public	325	30.12%
	Communist youth league member	481	44.58%
	Chinese communist party members	273	25.30%

### 5.2 Cronbach’s *α* coefficient analysis

First, we conducted Cronbach’s α coefficient analysis on the questionnaire data, with results presented in Table 2. Generally, a Cronbach’s *α* coefficient above 0.9 indicates excellent reliability of the test or scale; coefficients between 0.8–0.9 suggest moderate reliability, 0.7–0.8 indicate acceptable reliability, 0.6–0.7 represent average reliability, and 0.5–0.6 suggest suboptimal reliability. A coefficient below 0.5 warrants consideration of questionnaire redesign.

**Table 2:** Results of Cronbach’s *α* coefficient test.

Cronbach’s *α*	Standard Cronbach’s *α*	Sample number
0.850	0.856	1,079

As shown in Table 2, the Cronbach’s α coefficient of the questionnaire is 0.850, indicating excellent reliability.

### 5.3 Validity analysis

We conducted validity analysis on the data using KMO and Bartlett’s tests, with results presented in Table 3. Generally, factor analysis is considered highly suitable when KMO values exceed 0.9; moderate suitability ranges from 0.8 to 0.9; acceptable ranges from 0.7 to 0.8; acceptable ranges from 0.6 to 0.7; poor ranges from 0.5 to 0.6; and unsuitable ranges below 0.5. Table 3 shows that the data has a KMO value of 0.749 and a Bartlett’s sphericity test ***p-value of 0.000, indicating excellent questionnaire validity.

**Table 3:** Validity analysis results.

Measurement indicators		Actual value
KMO		0.749
Bartlett sphericity test	Approximate chi-square	163.317
	df	28
	*p*	0.000^***^

^***^Indicates a significance level of 1%.

### 5.4 Collinearity test

To assess potential collinearity issues in the data, we conducted linear regression analysis, with results presented in Table 4. The VIF (Variance Inflation Factor) indicates the severity of multicollinearity, serving as a key metric to evaluate whether the model exhibits high correlations among explanatory variables. Generally, VIF values should be below 10 or 5, with 5 being the strict threshold. An infinite VIF value (denoted as inf) suggests collinearity, necessitating further investigation. The results demonstrate that all VIF values remain below 5, indicating no multicollinearity issues and confirming a well-constructed model.

**Table 4:** Collinearity test results.

Item	VIF	*p*
Sex	1.12	*p* = 0.000^***^
Age	1.068	
Learning phase	1.016	
Political status	1.182	
AI precise feedback	1.897	
Intrinsic value identity	1.906	
Critical consciousness Transformation	2.234	

### 5.5 Path analysis

**Figure 2:**
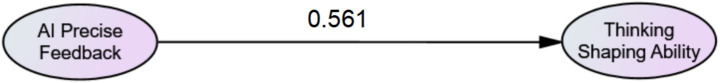
Path analysis modeling results.

This study employed path analysis to validate the direct effect hypothesis, specifically examining whether AI precision feedback significantly positively correlates with college students’ thinking shaping ability. During path model construction, AI precision feedback was treated as the independent variable, while thinking shaping ability served as the dependent variable, with direct analysis of their path coefficients. The modeling results are presented in Figure 2.

Furthermore, path analysis was employed to generate the influence path coefficient table (Table 5) and model fit index table (Table 6). The standardized coefficient of the AI Precision Feedback → College Students’ Thinking Shaping Ability path is 0.561, indicating strong influence. The C.R. value of 6.179 is significantly higher than 1.96, with a ***p-value of 0.000 at the 1% significance level, confirming a statistically significant positive correlation between AI Precision Feedback and College Students’ Thinking Shaping Ability. This validates *Hypothesis 1*. The model fit indices (GFI, RMR, CFI, NFI, and NNFI) all fall within acceptable ranges, demonstrating excellent model fit and further supporting *Hypothesis 1*.

**Table 5:** Path coefficient influence table.

X → Y	Non standardized coefficient	Standardized coefficient	S.E.	C.R.	*p*
AI precision feedback → college students’ thinking shaping ability	0.478	0.561	0.077	6.179	0.000^***^

^***^Represents significance levels of 1%.

**Table 6:** Model fitting indicators table.

Fitness index	Value range	Actual value	Fitting condition
GFI	>0.9	0.965	Accept
RMR	<0.05	0.013	Accept
CFI	>0.9	0.976	Accept
NFI	>0.9	0.937	Accept
NNFI	>0.9	0.940	Accept

### 5.6 Bootstrap sampling analysis

This study employs the Bootstrap sampling method to primarily analyze mediating effects, specifically to verify whether intrinsic value identification significantly mediates the relationship between AI precision feedback and college students’ thinking shaping ability. The Bootstrap method generates a large number of indirect effect estimates through repeated sampling, thereby constructing confidence intervals for these effects. This approach provides more accurate estimation and testing results for mediating effects, mitigates potential biases inherent in traditional methods, and enhances the reliability of research conclusions.

Using the Bootstrap sampling method to analyze the mediating effect, we obtained the results as shown in Table 7. The ***p-value of this mediating effect is 0.006, indicating significance at the 1% level. The 95% Bootstrap Confidence Interval (CI) ranges from 0.061 to 0.284, with no values within 0. This demonstrates that intrinsic value identification significantly mediates the relationship between AI precision feedback and college students’ thinking shaping ability, with a mediating effect value of 0.153 and a proportion of 32.01%. Thus, this confirms the validity of Research *Hypothesis 2*.

**Table 7:** Results of mediation analysis.

Path	*c* gross effect	*a*	*a* (*p*)	*b*	*b* (*p*)	a*b mediation effect size	a*b (Boot SE)	a*b (*z*)	a*b (*P*)	a*b (95% BootCI)	*c*’ direct effect	*c*’ (*p*)	Conclusion
AI precision feedback → intrinsic value identification → college students’ thinking shaping ability	0.478	0.5	0.000^***^	0.306	0.004^***^	0.153	0.054	2.84	0.006^***^	0.061–0.284	0.325	0.001^***^	Accept

^***^Represents significance levels of 1%.

### 5.7 Moderation effect analysis

The moderation effect test enables in-depth exploration of complex variable relationships, revealing the mechanisms of moderating variables and providing richer insights into how AI precision feedback shapes college students’ thinking shaping ability. This study employs the moderation effect test to examine *Hypothesis 3*, specifically verifying whether critical consciousness transformation moderates the relationship between intrinsic value identification and students’ thinking shaping ability.

**Figure 3:**
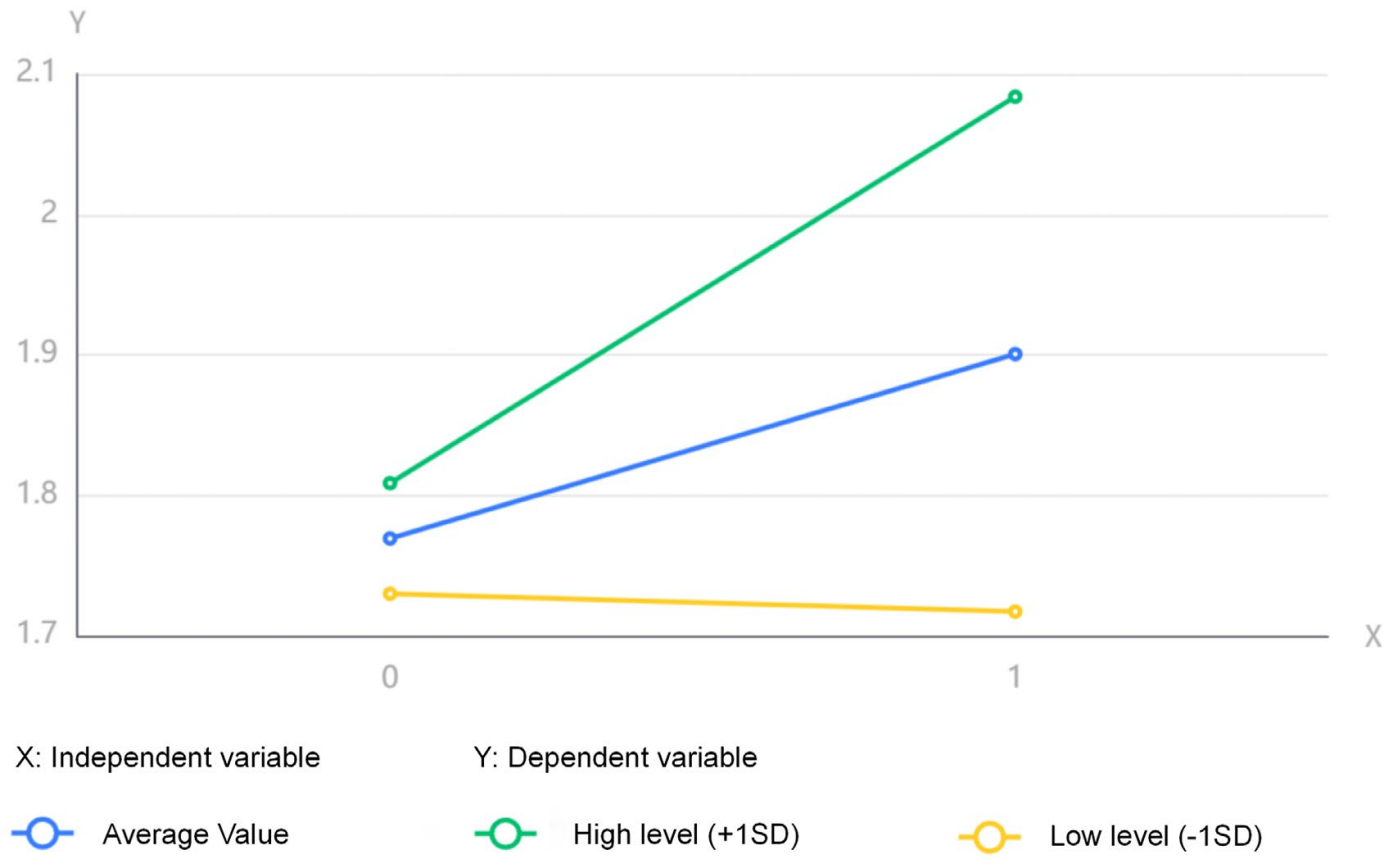
Simple slope diagram of regulation.

The moderating effect analysis yielded the following results (Table 8) and a simple slope plot (Figure 3). The table indicates that the interaction term ‘Intrinsic Value Identification*Critical Consciousness Transformation’ achieved a significant **p-value of 0.040, confirming the model’s validity. This suggests that the critical consciousness transformation significantly moderates the effect of Intrinsic Value Identification on college students’ thinking shaping ability.

**Table 8:** Results of adjustment effect analysis.

Item	Model 1				Model 2				Model 3			
	Coefficient	Standard error	*t*	*p*	Coefficient	Standard error	*t*	*p*	Coefficient	Standard error	*t*	*p*
Intrinsic value identification	0.514	0.091	5.677	0.000^***^	0.21	0.11	1.903	0.061^*^	−0.246	0.243	−1.011	0.315
Critical consciousness transformation					0.534	0.128	4.17	0.000^***^	0.049	0.264	0.187	0.852
Intrinsic value identification*Critical consciousness transformation									0.18	0.086	2.092	0.040^**^
*R* ^2^	0.285				0.412				0.443			

^***^, ^**^, and ^*^represent significance levels of 1, 5, and 10%, respectively.

Figure 3 reveals the magnitude differences in how Intrinsic Value Identification influences College Students’ Thinking Shaping Ability across three Critical Consciousness Transformation levels (low, medium, high). The significant variation in the independent variable’s effect on the dependent variable across these levels demonstrates that Critical Consciousness Transformation plays a significant moderating role.

Therefore, *Hypothesis 3* is confirmed.

## 6 Research conclusions

This study investigates how AI precision feedback shapes college students’ thinking shaping ability, with particular focus on the mediating role of intrinsic value identification and the moderating effect of critical consciousness transformation. The findings yield significant theoretical and practical implications.

First, AI precision feedback demonstrates a significant positive correlation with the development of college students’ thinking shaping ability. In the current era of digital transformation in education, traditional feedback models struggle to meet the evolving needs of students’ intellectual growth due to limitations like delayed responses and generalized approaches. AI-driven precision feedback, however, offers tailored guidance through data-driven personalization, real-time interaction, and targeted improvement suggestions. By continuously collecting and analyzing students’ learning data, it accurately identifies knowledge gaps and provides customized error correction recommendations. This enables students to promptly understand their academic progress, boost motivation, and effectively enhance thinking shaping ability. These findings provide compelling evidence for the effectiveness of AI applications in higher education, further advancing the integration of AI technologies into educational practices.

Second, intrinsic value identification plays a significant mediating role between AI precision feedback and college students’ thinking shaping ability. As a profound understanding and positive experience of learning activities and their significance, intrinsic value identification serves as the internal driving force for students’ proactive learning. When college students develop strong intrinsic value identification with AI precision feedback, they perceive it as an effective means to enhance learning outcomes and promote thinking shaping ability. This leads them to actively accept feedback information and internalize it as motivation and direction for their learning. They become more willing to learn and improve based on feedback, actively participate in learning activities, and thereby foster the development of cognitive shaping abilities. This discovery reveals the underlying psychological mechanism through which AI precision feedback influences college students’ thinking shaping ability, providing crucial theoretical support for improving the effectiveness of AI education applications. In educational practice, educators can guide students to recognize the value of AI precision feedback, enhance their intrinsic value identification with feedback, and thus better leverage its promoting role in thinking shaping ability.

Third, critical consciousness transformation plays a moderating role between intrinsic value identification and college students’ thinking shaping ability. In today’s era of information explosion and rapid technological advancement, students are no longer passive recipients of AI-generated feedback. As the ability to filter, question, integrate, and apply external information, critical consciousness transformation helps students rationally evaluate the advantages and limitations of intelligent feedback, avoiding blind reliance or excessive rejection. Research reveals significant differences in how students with varying levels of critical consciousness transformation perceive the impact of intrinsic value identification on thinking shaping ability. Those with higher critical consciousness transformation demonstrate stronger analytical judgment of AI feedback, effectively utilizing it to enhance their thinking. Conversely, students with lower levels may struggle to fully leverage intrinsic value identification’s positive influence on thinking shaping ability. These findings underscore the importance of cultivating critical consciousness transformation in education, emphasizing the need to guide students in developing critical thinking skills. This enables them to properly utilize AI’s precise feedback in complex information environments, achieving effective thinking shaping ability.

This study not only enriches the theoretical framework of AI education applications, providing valuable references for future research, but also offers targeted guidance for higher education practices. Universities and educators should fully recognize the crucial role of AI precision feedback in shaping college students’ thinking patterns. By actively integrating AI educational technologies while cultivating students’ intrinsic value identification and critical thinking transformation capabilities, we can comprehensively enhance their cognitive literacy and comprehensive competencies, thereby laying a solid foundation for their future development.

## 7 Research significance and innovations

### 7.1 Theoretical implications

Firstly, this study expands the theoretical boundaries of AI education applications. Moving beyond existing literature that primarily focuses on AI feedback’s impact on explicit outcomes such as academic performance and knowledge acquisition, we extend our research perspective to the implicit competency dimension of cognitive shaping ability, revealing the profound connection between technological interventions and higher-order thinking development. By constructing a mediating mechanism model of “AI precision feedback → intrinsic value identification → cognitive shaping ability,” this research provides a novel theoretical framework for understanding the “black box” of technology-enabled education, enriching the theoretical construction of “technology-cognition” relationships in educational technology studies.

Second, this study deepens the theoretical understanding of intrinsic value identification in digital learning contexts. By applying traditional intrinsic value identification theory to AI education scenarios, we propose a three-dimensional framework comprising utility identification, process identification, and self-connection identification. The research also identifies emerging influencing factors such as technological trust and de-evaluation anxiety effects, thereby expanding the application boundaries of Expectancy-Value Theory in technology-mediated learning environments. These findings provide a novel analytical framework for motivation theory research in the digital era.

Third, this study validates the cross-context applicability of the moderated mediation model. Through empirical verification, we confirm the theoretical proposition that “mediating effects are influenced by moderating variables” holds true in the field of AI education. This provides a methodological framework for analyzing complex educational phenomena through multi-mechanism approaches, facilitating the paradigm shift in educational research from “binary relationships” to “multi-factor mechanisms.”

### 7.2 Practical implications

Firstly, the findings of this study provide actionable guidelines for teachers to integrate AI feedback with traditional teaching methods. The research highlights the critical mediating role of intrinsic value identification, suggesting that educators should focus on the following key aspects when designing AI-assisted instruction: Pre-intervention value orientation—clearly articulating the specific cognitive development benefits of AI tools before implementation; Process-oriented experiential reinforcement—designing low-threshold, high-success-rate AI interaction tasks to accumulate positive learning experiences; Reflective meaning construction—facilitating student sharing of AI feedback experiences to elevate individual insights into collective consensus. This “guidance-experience-reflection” three-phase model can be directly applied in classroom practice.

Second, it provides precise entry points for cultivating critical thinking among college students. Moderation effect analysis demonstrates that the transformation of critical awareness significantly enhances the practical outcomes of value identification. Universities should incorporate “algorithmic literacy” into general education systems and improve students ‘critical awareness transformation capabilities through the following approaches: Cognitive level—Offering AI principles courses to deconstruct algorithmic logic; Skill level—Designing specialized “AI feedback evaluation” training programs to develop students’ abilities in questioning, verifying, and integrating information; Attitudinal level—Creating open and inclusive classroom cultures that encourage students to challenge AI feedback and justify its rationality.

Third, it provides demand-driven guidance for iterative upgrades of intelligent education products. Research findings offer critical insights for educational technology enterprises: The design of AI feedback systems should shift from “knowledge-oriented” to “thinking-oriented” approaches, incorporating functional modules such as visualization of cognitive processes, multi-path comparison presentation, and metacognitive prompts. Simultaneously, systems should integrate “critical use guidance mechanisms,” including regular reminders about “AI feedback limitations” and predefined “manual review trigger conditions,” to mitigate over-reliance risks at the technical architecture level.

### 7.3 Research innovation

First, Perspective Innovation: Paradigm Shift from “Technical Efficiency” to “Development Mechanisms.” Unlike existing studies focusing on validity validation of “AI educational application efficacy”, this research emphasizes mechanism analysis of “how AI precision feedback influences cognitive shaping capabilities”. For the first time, it systematically reveals the transmission effect of intrinsic value identification and boundary effects of critical consciousness transformation, establishing a complete explanatory chain of “technical input → psychological processing → capability output”. This advancement propels AI education research from “effect evaluation” to “process comprehension”, marking a significant shift in scholarly focus.

Second, theoretical integration and innovation: interdisciplinary convergence of theories. This study achieves three levels of theoretical integration: the fusion of educational technology (AI-based precision feedback) and learning science (intrinsic value identification) to reveal psychological mediation mechanisms of technological interventions; the integration of motivation theory (expectancy theory) with critical pedagogy (critical consciousness transformation) to elucidate the interaction between value internalization and rational reflection; and the combination of cognitive psychology (cognitive shaping) with algorithmic sociology (algorithmic literacy) to address core issues in contemporary philosophy of technology. This interdisciplinary approach transcends the explanatory limitations of single-theory perspectives, significantly enhancing the theoretical depth of the research.

Third, context-focused innovation: A practical response to the generative AI era. This study directly addresses educational transformations driven by the widespread adoption of generative AI systems like ChatGPT, framing its research context within the novel “college student-AI feedback system” interaction model. It explores scientific issues underlying real-world concerns such as algorithm dependency and cognitive regression. The findings provide forward-looking insights into reshaping education in the generative AI era, offering empirical support for building a new educational ecosystem characterized by human-machine collaboration.

## 8 Research limitations and future research directions

### 8.1 Research limitations

First, limitations in sample selection. This study involved 1,079 college students primarily from comprehensive universities in China. The sample size is relatively small, and differences in cognitive training models across disciplines and AI application scenarios were not explored. Additionally, strict stratification control was not implemented regarding grade distribution, and the impact of academic year differences on AI feedback acceptance was inadequately examined, which may limit the external validity of conclusions. Second, limitations in measurement tools. Data collection relied on self-administered questionnaires that, despite pre-revised versions, still exhibit shortcomings: AI precision feedback assessment focuses on subjective perceptions (e.g., timeliness and personalization) rather than direct collection of backend behavioral data from learning platforms (e.g., feedback click-through rates, dwell time, follow-up improvement behaviors), potentially creating discrepancies between subjective perceptions and objective usage patterns. Cognitive shaping ability, as a higher-order cognitive skill, cannot be fully captured through self-reporting alone. Future research should incorporate multi-method validation approaches such as behavioral experiments and project analysis for triangulation. Third, limitations in study design. This study employed a cross-sectional design, collecting all variable data at a single time point, which makes it difficult to rigorously establish causal temporal relationships between variables. Although statistical methods support testing for mediating and moderating effects, the causal chain of “AI precision feedback → intrinsic value identification → cognitive shaping ability” still requires longitudinal tracking studies or experimental designs for confirmation. Additionally, the study failed to control for potential confounding variables such as students’ prior cognitive abilities, disciplinary backgrounds, and AI usage experience, which may correlate with core variables and affect the accuracy of effect estimation. Fourth, limitations in situational control. The study did not differentiate between specific types of AI feedback (e.g., intelligent tutoring systems, automatic essay grading systems, programming error correction tools). Significant differences exist in information presentation methods, interaction depth, and feedback granularity across different AI feedback types, suggesting heterogeneous mechanisms of influence. Furthermore, the study did not distinguish contextual factors such as course settings (required/elective), task types (routine assignments/innovative projects), and teacher support levels, whose moderating effects on main effects require further exploration.

### 8.2 Future research directions

First, expand research samples to enhance external validity. Future studies should incorporate interdisciplinary perspectives to explore analytical differences across disciplines and majors, while including research from diverse university types to validate cross-institutional applicability of conclusions. Second, increase data sample sizes to confirm the generalizability of findings. Second, innovate research methodologies for multi-faceted validation. Future studies are recommended to adopt the method triangulation strategy. Behavioral data mining: Collaborate with learning platforms to collect process data including student clickstream patterns, eye-tracking metrics, and keyboard records to objectively characterize AI feedback usage patterns. Experimental design: Conduct randomized controlled trials to manipulate AI feedback presentation modes (immediate/deferred, detailed/abbreviated, personalized/standardized) and examine causal effects of design elements. Learning analytics: Develop predictive models to forecast students’ cognitive development levels based on historical data, enabling targeted interventions. Third, deepen mechanism exploration and refine theoretical models. Future research could conduct longitudinal tracking studies incorporating multiple mediating variables, examining cognitive load, metacognitive strategies, and learning engagement to construct and compare competitive mediation models. Simultaneously, expand the types of moderating variables by including technical characteristics (e.g., system transparency, naturalness of human-computer interaction), situational factors (e.g., teacher support, peer collaboration), and individual variables (e.g., cognitive styles, technological anxiety) to develop multi-level moderation effect models. Fourth, focus on specific contexts for detailed research. Future studies should further investigate generative AI scenarios using tools like ChatGPT and DeepSeek to analyze the unique impact of “generative feedback” (e.g., writing suggestions, code optimization) on creative thinking. Additionally, virtual reality simulations could be employed to explore how embodied characteristics of AI feedback in immersive VR environments influence spatial and design thinking mechanisms.

## Data Availability

The original contributions presented in the study are included in the article/supplementary material, further inquiries can be directed to the corresponding author.
